# Cellular Growth Arrest and Persistence from Enzyme Saturation

**DOI:** 10.1371/journal.pcbi.1004825

**Published:** 2016-03-24

**Authors:** J. Christian J. Ray, Michelle L. Wickersheim, Ameya P. Jalihal, Yusuf O. Adeshina, Tim F. Cooper, Gábor Balázsi

**Affiliations:** 1 The University of Texas MD Anderson Cancer Center, Department of Systems Biology, Houston, Texas, United States of America; 2 Center for Computational Biology, University of Kansas, Lawrence, Kansas, United States of America; 3 Department of Molecular Biosciences, University of Kansas, Lawrence, Kansas, United States of America; 4 SASTRA University, Tirumalaisamudram, Tamil Nadu, India; 5 Department of Biology and Biochemistry, University of Houston, Houston, Texas, United States of America; 6 Laufer Center for Physical & Quantitative Biology and Department of Biomedical Engineering, Stony Brook University, Stony Brook, New York, United States of America; University of Illinois at Urbana-Champaign, UNITED STATES

## Abstract

Metabolic efficiency depends on the balance between supply and demand of metabolites, which is sensitive to environmental and physiological fluctuations, or noise, causing shortages or surpluses in the metabolic pipeline. How cells can reliably optimize biomass production in the presence of metabolic fluctuations is a fundamental question that has not been fully answered. Here we use mathematical models to predict that enzyme saturation creates distinct regimes of cellular growth, including a phase of growth arrest resulting from toxicity of the metabolic process. Noise can drive entry of single cells into growth arrest while a fast-growing majority sustains the population. We confirmed these predictions by measuring the growth dynamics of *Escherichia coli* utilizing lactose as a sole carbon source. The predicted heterogeneous growth emerged at high lactose concentrations, and was associated with cell death and production of antibiotic-tolerant persister cells. These results suggest how metabolic networks may balance costs and benefits, with important implications for drug tolerance.

## Author Summary

In bacteria, changes in gene expression, with resulting changes in protein concentration, can drastically change how fast cells and cellular populations grow. This fact has big implications for how we treat infectious disease, which types of organisms make up our microbiomes, and what patterns of gene regulation have undergone evolutionary selection. Here, we show how, in principle, the expression level of a single enzyme can affect bacterial population growth by creating a threshold where cells grow optimally fast just below it, but rapidly reach a state of no growth just above it because metabolic byproducts build up and halt growth. The narrow margin between these two states makes entering either of them possible for the same bacterium because of intrinsic uncertainty, or "noise", in gene expression. The predicted result is a variety of growth rates in a single population of genetically identical cells, manifested as a mix of fast- and slow-growing cells. We created laboratory conditions that reproduce the effect in the model organism *E*. *coli*, and showed that there may be a benefit to having slower growing cells, because they can survive antibiotic exposure for longer.

## Introduction

Metabolism in single-celled organisms is subject to dynamic regulatory responses balancing cost and benefit [1–11]. Most single-celled organisms are under strong selective pressure to optimize growth in many conditions [12–23]. Nevertheless, some conditions exist where side effects of processing available metabolites slow growth. For example, mutations or rapidly changing conditions can result in toxic metabolic effects, including substrate-accelerated toxicity or even cell death. Examples include buildup of galactose derivatives arising from mutations in the Leloir pathway [24] and the lactose killing effect in bacteria [24–28] and yeast [29–31]. In the latter, byproducts of proton-catabolite symport in Major Facilitator Superfamily (MFS) permeases [32] are toxic [26, 33]. Defects in permease selectivity can result in excess sugar uptake causing excessive intracellular osmotic pressure [34].

Effects of metabolic stress have typically been considered at the population level, but recent findings suggest it may be important to consider the possibility that stress drives non-genetic variation between cells within a population. For example, in bacteria, metabolic starvation stress can induce toxin-antitoxin (TA) systems and resultant formation of non- or slow-growing antibiotic tolerant persister cells [35, 36]. This suggests a metabolic route to regulating cellular toxicity and growth arrest and raises the question of how metabolic cost and benefit affect population growth dynamics in the face of heterogeneity. In yeast, a thresholding effect in metabolism creates coexisting subpopulations of cells with different growth rates [31, 37]. Some signaling [38] and metabolic [39] pathways may have evolved to minimize intracellular noise of relevant protein or metabolite levels. However, single cells cannot control extracellular perturbations, and intracellular noise control is costly [40]. Multiple metabolic pathways in *Escherichia coli* have been found to operate close to the saturation point of their constituent enzymes [41], near an ultrasensitive threshold [42]. Beyond the threshold, intracellular metabolite concentrations rise sharply [42]. These studies suggest an important effect of intrinsically variable cellular metabolic states on cellular and population growth rates in the face of metabolic toxicity. However, the dynamics of cellular growth around metabolic thresholds is unknown.

In this study we developed mathematical and computational models of simple metabolic pathways. We determined the effects of pathway efficiency, cost, and demand on emergent cellular and population scale growth rates. Our results imply that, past a certain threshold, intracellular metabolite toxicity dominates growth kinetics, causing some cells to enter into a growth-arrested state while the rest of the population maintains fast growth. By growing *E*. *coli* cultures in varying levels of lactose, we confirmed cellular growth rate heterogeneity as theory predicts. Populations grown in conditions that produced a growth-arrested fraction of cells also had high frequencies of dead and antibiotic-tolerant persister cells. We propose a conceptual framework for understanding growth heterogeneity among individual cells as a consequence of optimizing population growth.

## Results

**Fig 1:**
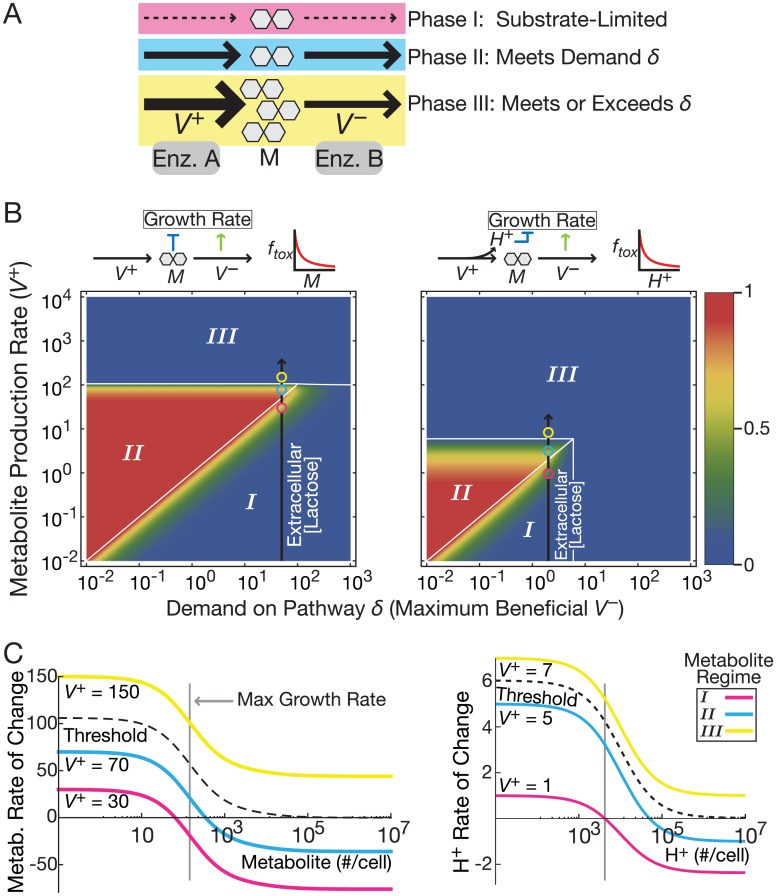
Three discrete growth phases in metabolic pathways. (A) A metabolic pathway consists of enzymes *A* and *B* that produce and consume the intracellular metabolite *M*, respectively. (B) Mathematical models predict three growth phases for combinations of metabolite production rate and demand *δ*. Color bar indicates normalized growth rate. Colored spots correspond to the colored lines in panel C. The black line represents the effect of experimental conditions changing extracellular lactose concentrations in *E*. *coli* causing intracellular lactose concentration changes because of LacY activity. Results are for two models of toxicity: metabolite buildup (left) and permease proton symport (right). (C) Mathematical models predict that growth is maximized below a threshold production rate (dashed line between cyan and yellow) past which no steady state exists. Increasing the rate of metabolite production (*V*^+^) translates the rate curves upward. When the rate is beyond the dashed line, there is a runaway buildup of metabolite and consequent toxic effects. Results are for two models of toxicity: metabolite buildup (left) and permease proton symport (toxic byproduct; right). The toxic byproduct model has two variables; we plot only the rate of toxic byproduct buildup for simplicity because it crosses the threshold at a lower *V*^+^.

We consider a generic model for an irreversible metabolic pathway with enzymes *A* and *B* producing and consuming an intracellular metabolite *M* with fluxes *V*^+^ and *V*^*–*^, respectively (Fig 1a). *M* is also degraded by first-order dilution from cellular growth. This model captures key aspects of single metabolite conversion steps. We use the model to examine the effect of metabolic conditions at various time and size scales: short timescales (faster than gene regulation), intermediate timescales on the order of gene regulatory events, and the larger size and timescale of population growth.

### Cost-benefit models on short timescales

To study the consequences of metabolic cost/benefit trade-offs on short timescales, we modeled metabolite levels with constant enzyme concentrations. Enzyme *B* supports growth via the flux *V*^*−*^ relative to a demand, *δ*, that determines the flux optimizing cell growth. Cost, or toxicity, may arise from the substrate, *M*, or from metabolic byproducts (e.g., symported protons from a permease protein). Increasing metabolite production flux relative to demand (*V*^+^/*δ*) speeds cellular growth when *V*^+^ is not too high (Fig 1a and 1b). However, for excessively high *V*^+^ (Equation S8 in S1 Text) the consumption flux *V*^*−*^ saturates. In this regime, our theory predicts an increase in the concentration of *M* (or byproducts of *M* production) until growth stalls (Fig 1c).

Consequently, we predict three qualitatively distinct cellular growth regimes with this model (Fig 1b and 1c). Regime I corresponds to substrate-limited starvation in which low metabolite production limits growth. In Regime II (satiation), metabolite production meets demand for the pathway while toxicity is low enough not to drive cells into growth arrest. Regime III (surfeit) represents a phase where metabolite production exceeds the demand and metabolic benefit cannot compensate for toxicity, resulting in stalled growth and possibly cell death. Even slight toxicity from pathway activity and saturability of the consumption flux *V*^*−*^ are sufficient for the emergence of Regime III (S1 Text; S1 Fig).

For moderate pathway demand, low total flux suffices to meet the demand. Thus, cells remain well below toxic levels of activity and Regime II is wide (Fig 1b). On the other hand, for high pathway demand, the fastest cellular growth rate is near the point where Regimes I, II and III converge (Fig 1b). For demand higher than this convergence point, no level of metabolic flux can offset the costs of toxicity. For demand just below the convergence point, stochastic fluctuations in enzyme levels could drive fast-growing cells to drastically slow the cellular growth rate. Stochastic crossing of the critical surface bordering Regime III soon becomes irreversible at least until other compensating mechanisms, such as toxin efflux, ensue (Fig 1c). This happens because the stabilizing effect of growth- or enzyme-mediated dilution of toxicity is absent in Regime III, causing continued toxic buildup. As long as the probability of entering Regime III is non-zero, biochemical irreversibility causes any particular cell to eventually end up in the growth-arrested state. Yet, if cell division is sufficiently faster than the rate of threshold crossing, the population can still grow even while individual cells arrest their growth.

In the presence of metabolic fluctuations, the existence of a regime characterized by growth arrest suggests two alternative strategies to optimize population growth when demand for the pathway product is high. First, all cells could “play safe”, avoid Regime III and support relatively uniform growth rates across the cell population by ensuring low production or high consumption of the metabolite. However, limiting metabolite production causes starvation and slows growth, while excessive, underutilized downstream metabolic flux capacity incurs a cost from enzyme expression [25]. Alternatively, metabolic pathways could operate close to the critical threshold, maximizing cell population growth rates, but also risking stochastic transitions into Regime III and subsequent growth arrest. We hypothesized that sufficiently high growth rates of a metastable, sub-threshold cell subpopulation can more than offset losses across the threshold. This would lead to faster population growth than the strategy of uniform cellular growth rates achieved by lowering the ratio of metabolite production to consumption. Consequently, we predict that a signature of cell populations near the border of Regime III will be growth rate heterogeneity as a result of noise causing some cells to exceed the threshold (fitness noise [43]). Such populations would consist of a rapidly dividing majority of cells that constantly supply a slower-growing subpopulation prone to entering growth arrest. If the cells move further toward Regime III, population growth rate should slow, giving rise to an optimum slightly below Regime III.

### Growth rate heterogeneity in single-cell and population models

**Fig 2:**
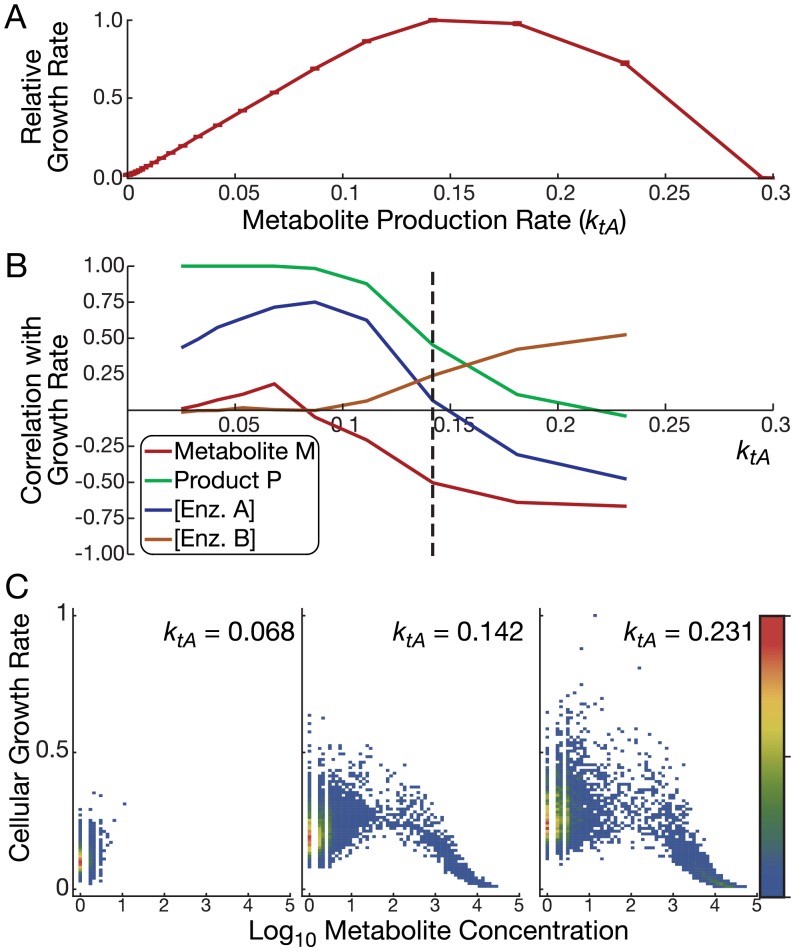
Predicted population growth rates for varying substrate import rates in simulated metabolic pathway models. (A) Average single cell growth rates predicted with a stochastic model (*N* = 10,000 trajectories) with substrate toxicity. Intracellular metabolite production rates are controlled by changing the transcription rate of Enzyme A (*k*_*tA*_). The discrepancy with experimentally measured growth rates at high metabolite concentrations (Fig 3a) are discussed in Methods. (B) Correlations between single-cell molecule numbers and growth rates reveal mechanisms of growth arrest in stochastic simulations. Positive correlations imply that the molecule species improves growth, while negative correlations imply the opposite. The black dashed line indicates the parameter value predicted to give peak growth rate. Correlations where all trajectories enter growth arrest (making model predictions irrelevant) are not shown. (C) Predicted population densities (color bar) of cellular growth rate and metabolite from stochastic simulations in the low toxicity case. All trajectories in these three cases start with low metabolite concentrations. As the simulations proceed, trajectories exceeding a threshold concentration of metabolite transition from the low-metabolite subpopulation to the high-metabolite subpopulation. The predicted growth rate of the high-metabolite subpopulation decreases as metabolite continues to build up.

We next computationally tested our hypothesis in models of cellular growth while accounting explicitly for gene expression, as well as biochemical, noise. We used stochastic simulations to capture the stimulatory and inhibitory effects of pathway activity on cellular growth, molecular dilution, and gene transcription. By simulating multiple trajectories, we predicted average cellular growth rates as a representation of population growth rates (Fig 2a–2c; Table 1; parameters *k*_*tA*_ and *k*_*tB*_ are represented by *k*_*tE*_ in the table; see footnote † in Table 1). We therefore relaxed the assumption that metabolite dynamics are faster than gene expression dynamics, but still assumed metabolic cost and benefit to arise quickly from the metabolic intermediate and product, respectively. We scanned the rate of metabolite production by changing the transcription rate of enzyme *A* and performing N = 10,000 simulations for each value.

**Table 1:** Reactions, propensities and parameter values used for stochastic simulations.

Reaction number	Reaction*	Propensity^†^	Parameter values^‡^
1	→ *mRNA*_*E*_	*k*_*tE*_ *f*_*tox*_ *f*_*gr*_ g*	0.05 s^-1^
2	*mRNA*_*E*_ → Ø	*k*_*mdeg*_ *mRNA*_*E*_	0.0033 s^-1^
3	→ *E*	*k*_*tsn*_ *mRNA*_*E*_	0.05 s^-1^
4	*E* → Ø	*k*_*g*_ *f*_*tox*_ *f*_*gr*_ *E*	0.0006 s^-1^
5	*g* ⇌ g*	*k*_*gon*_ *g*, *k*_*goff*_ g*	*k*_*gon*_ = 0.00045 s^-1^ *k*_*goff*_ = 0.0028 s^-1^
6	*A + S ⇌ A*.*S*	*k*_*im*_ *A*, *k*_*–1*_ *A*.*S*	[10^−5^, 10] s^-1^, 2 s^-1^
7	*A*.*S → A + I*	*k*_*cat1*_ *A*.*S*	1 s^-1^
8	*B + I ⇌ B*.*I*	*k*_*2*_ *B·I*, *k*_*–2*_ *B*.*I*	2 Ω/(#×s), 2 s^-1^
9	*B*.*I → B + P*	*k*_*cat2*_ *B*.*I*	1 s^-1^
10	*A*.*S* → Ø	*k*_*g*_ *f*_*tox*_ *f*_*gr*_ *A*.*S*	0.0006 s^-1^
11	*B*.*I* → Ø	*k*_*g*_ *f*_*tox*_ *f*_*gr*_ *B*.*I*	0.0006 s^-1^
12	*I* → Ø	*k*_*g*_ *f*_*tox*_ *f*_*gr*_ *I*	0.0006 s^-1^
13	*P* → Ø	*k*_*u*_ *P*	2 s^-1^

*Variable definitions: *E*: any enzyme; *A*, *B*: specific metabolic enzymes; *S*: substrate; *I*: intermediate metabolite; *P*: product. There is one mRNA variable for each enzyme.

^†^Unbound substrate *S* is held constant and subsumed into parameter *k*_*1*_. Dilution rate propensities are multiplied by function *f*_*tox*_ and *f*_*gr*_ taking the same mathematical form as Equations S2 and S4 in S1 Text. *k*_*tE*_ takes separate values *k*_*tA*_ and *k*_*tB*_, and *k*_*tA*_ is varied to change the expression ratio.

^‡^Ω represents cell volume. Parameter *k*_*im*_ is a composite parameter equal to [metabolite]×*V*_*i*_ where units of *V*_*i*_ are Ω/(#×s); *k*_*im*_ is varied between the values 10^−5^ and 10 in Fig 2. In the low toxicity case, we have *θ* = 10,000; in high toxicity, *θ* = 1. Demand parameter *δ* = 400. Parameters are based on previous estimates [39]. Unlike the previous model [39], *k*_*g*_ represents the maximum attainable growth rate so the value is higher.

Consistent with the simpler models, a peak population growth rate occurs at intermediate metabolite production rates (Fig 2a). Complete growth arrest predicted at high metabolite production rates relates to the lack of population dynamics in stochastic simulations (Methods).

Correlations between variables in the simulation and growth rates show the predicted effect of each component on growth (Fig 2b). Namely, positive correlations imply that the molecule species improves growth, while negative correlations imply the opposite. Below peak population growth, single-trajectory (corresponding to single-cell) growth rate predictions were positively correlated with enzyme *A*, which increases metabolite levels (blue line, Fig 2b). Above peak population growth, the growth rate predictions correlated negatively with enzyme *A*, but correlated positively with enzyme *B* (orange line, Fig 2b). Above starvation levels of metabolite production rate, the amount of metabolite *M* was negatively correlated with predicted growth rates (red line, Fig 2b). These results confirm the same mechanism driving toxicity in this model as the simpler model presented in Fig 1.

We then compared cellular growth rates to metabolite concentrations in individual cell simulations (Fig 2c). Red regions depict the most frequent simulation outcomes, while blue shows ones. Uncolored areas show regions with no simulation trajectories. Gaps in the density at low metabolite concentration correspond to low metabolite levels (low integer values, such as 1–2 per cell). These plots show that population-level growth at various metabolite production rates arises from individual cells being in qualitatively distinct growth regimes. Most trajectories predict low metabolite levels. At higher metabolite production rates, a fraction of cells enter the growth-arrested phase because of high metabolite concentrations. We analyze the implications of these single-cell predictions on population dynamics in S1 Text and S3 Fig. Together, the single-cell and population-scale models provide a consistent prediction that metastable population dynamics can cause a non-monotonic relationship between metabolite levels and growth rates.

### Fitness trade-off for *E*. *coli* in lactose

To experimentally test which population-level growth strategy is followed by a fast-growing bacterial population with high metabolite demand, we exploited *E*. *coli* lactose catabolism. In this pathway, imbalances between metabolite production and consumption are toxic, and there is the potential for growth modulation via downstream events in glucose and galactose catabolism. Lactose permease LacY (equivalent to enzyme *A* in Fig 1a), catalyzes metabolite production by importing extracellular lactose. The consumption flux consists of lactose conversion into glucose and galactose by β-galactosidase LacZ (equivalent to enzyme *B* Fig 1a). Excess lactose in growth media may thus inhibit growth via mechanisms related to lactose killing or buildup of one or more metabolites. Nevertheless, intracellular lactose and subsequent processing of its catabolic products is necessary for cellular growth when no other carbon source is present. Thus, the system has a trade-off with any toxic effects that may arise.

**Fig 3:**
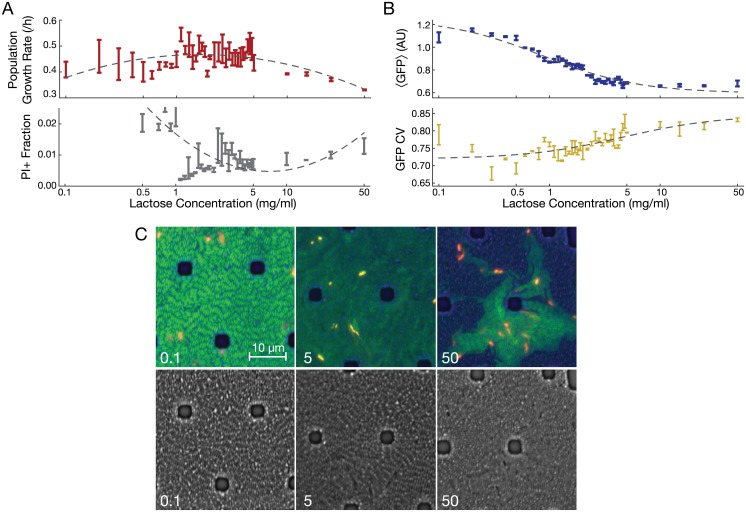
Population and individual cell fitness of *E*. *coli* (*lacI*^−^ B REL606) in varying growth conditions. (A) Mean ± SEM (*N* = 3) growth rate (*p* < 10^−6^ for no trend in lactose concentrations > 1 mg/ml) and fraction of propidium iodide-stained (PI+) cells (*p* = 0.0051 for no PI+ trend in lactose concentrations > 1 mg/ml) at various lactose concentrations. Dashed lines indicate quadratic regression models, which fit significantly better than do linear models (see text for details). PI+ fractions at low lactose concentrations are shown in S4c Fig. (B) Mean ± SEM (*N* = 3) expression of GFP at various lactose concentrations (*p* = 0.00016 for no trend). CV, coefficient of variation (*p* < 10^−6^ for no trend). Dashed lines indicate fits of statistical models used as a guide to the eye (fitted model is: $y=a+\frac{b}{c+x}$). (C) PI-stained *E*. *coli* grown in a microfluidic device perfused with the indicated concentration of lactose (mg/ml). Note the patchy distribution of fast growing (low GFP) and slow- or non-growing (high GFP) cells at 50 mg/ml lactose. Brightfield alone is shown below. PI staining identifies dead cells and appears red or yellow, depending on the amount of GFP in the same cell. Dark spots are silicone support structures.

We examined the effect of extracellular lactose concentration on the population growth rate of *E*. *coli* in simple settings devoid of feedback regulation. To do so, we grew a *lacI*^−^ strain derived from REL606 [44] at increasing lactose concentrations (corresponding to increasing flux *V*^+^ along the black lines in Fig 1b). Population growth rates were highest at intermediate lactose concentrations (1–5 mg/ml; Fig 3a). A quadratic model (dashed line in Fig 3a; Akaike information criterion [AIC] = -148.674) with a growth rate optimum at intermediate lactose concentration describes the pattern better than a linear model (AIC = -129.603). Parallel growth experiments on the ancestral *lacI*^wt^ strain confirmed the same trend (S4a and S4b Fig). These results are consistent with our prediction that toxicity of high lactose concentrations will lower population growth rates in cultures acclimatized to those conditions. However, they cannot distinguish between uniform toxicity to the entire population and increased growth heterogeneity with a subset of cells transitioning into growth arrest or death. To distinguish between these two possibilities we used flow cytometry and microscopy to characterize growth properties in single cells.

### Cellular growth heterogeneity and killing in lactose

To determine whether population growth rates were characterized by uniform or heterogeneous cellular growth, we used a chromosomally-integrated *P*_*lacO1*_-GFP reporter (with constitutive expression in the *lacI*^−^ strain [44]) as a single cell-level growth rate sensor (Fig 3b). To calibrate the sensor, we matched the demonstrated negative dependence of constitutively expressed protein concentrations on bacterial growth rates [45] to our data. That is, we took lower fluorescence readouts to indicate faster growth. Low mean fluorescence values observed at high lactose concentrations indicate fast growth of single cells, yet the population growth rate is low in those conditions. We compared the likelihood that two possible models explain the results. In one, we assumed balanced exponential growth (uniform growth model). In the other, we added a mechanism for lactose-dependent transition into a non-growing state in which the mean fluorescence is determined by the majority of growing cells (heterogeneous growth model). We found that the model incorporating heterogeneous growth explained the pattern of mean fluorescence much better than the model enforcing uniform growth (S5 Fig). Moreover, fluorescence heterogeneity (CV) increased significantly as lactose doses increased (Fig 3b), as did skewness and kurtosis (S6 Fig). These increases indicate both an increase in the relative heterogeneity of growth rates and fatter tailed growth rate distributions at high lactose concentrations.

Cell death may be a feature of cell sub-populations following prolonged time in regime III. To estimate cell death in each condition, we used propidium iodide (PI) staining to identify dead cells following incubation at different lactose concentrations. As predicted, we saw a significant increase in dead (PI+) cells as lactose concentrations increased past the point supporting a maximum growth rate (Fig 3a). This result is consistent with metabolite toxicity concurrent with growth arrest resulting in an increased chance of cellular death. We also observed an elevated frequency of PI+ cells in low lactose, likely attributable to starvation-induced cell death [46] (S4c Fig). To further confirm the pattern of PI staining, we fit the data with a quadratic curve (Fig 3a, dashed line); AIC = -694.25 vs -655.964 for a linear model.

In the above experiments we used shaking microplates that provide less aeration than some other methods, and did not permit time-lapse detection of fluorescence. We therefore next visualized the competing effects of growth stimulation and inhibition on individual cells at different lactose concentrations. We did so with time-lapse fluorescence microscopy of *lacI*^−^*P*_*lacO1*_-GFP *E*. *coli* cells growing in an incubated microfluidic device with a constant flow of freshly oxygenated air over the cell growth chamber (Fig 3c, S1–S4 Movies). After growth for 18–24 hours, we stained for dead cells using 1 μg/ml PI in the perfused medium. The resulting images show striking qualitative differences in population structure. In low lactose (0.1 mg/ml), cells exhibited uniform green fluorescence (and therefore, growth rates). At 50 mg/ml lactose, clustered subsets of cells exhibiting higher fluorescence emerged. These patterns are consistent with subpopulations once poised on the threshold between fast growth and growth arrest that subsequently stopped growing (Fig 3c). In 50 mg/ml lactose, PI-stained cells co-localized with these islands of high fluorescence, corresponding to an elevated chance of cell death arising from growth-arrested subpopulations.

Taken together, these observations support a model of population growth balancing the costs and benefits of lactose metabolism. There is a limit to the benefit of lactose in the media, and growth is inhibited in a fraction of cells as the concentration of lactose increased. Metabolite buildup may also occur downstream in the pathway, in which case enzymes *A* and *B* are downstream as well, or upstream, due to LacY saturation or toxicity from some off-target mechanism. We discuss why the latter possibility is unlikely in S1 Text and Discussion. Because we observed acclimatized cultures in constant growth conditions, the underlying mechanism for growth inhibition at high concentrations is a continuously occurring process, as opposed to a shock caused by changing growth conditions.

### Growth-arrested cells correlate with antibiotic persisters

**Fig 4:**
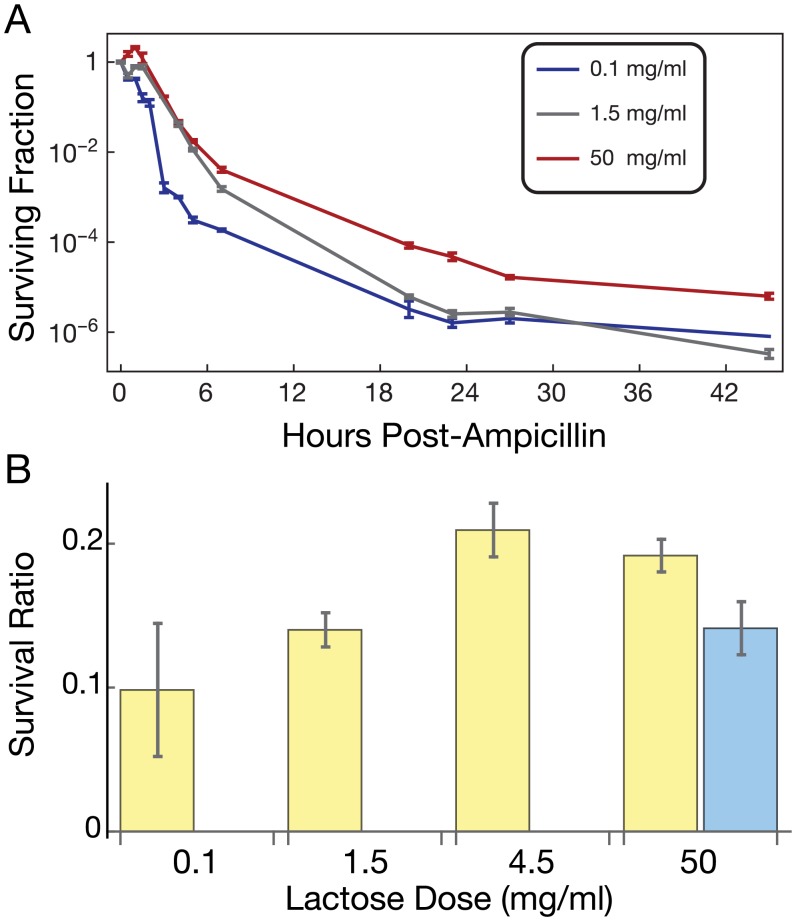
Protection from antibiotics in growth-arrest-prone media. (A) Survival curves of bacteria grown at indicated lactose concentrations in ampicillin-treated cultures (100 μg/ml). (B) Culture conditions favoring growth arrest enhance the presence of antibiotic tolerant cells after 20 h treatment of 32 μg/ml doxycycline (blue bars; ANOVA *p* = 0.003) or 100 μg/ml ampicillin (yellow bars; ANOVA *p* = 0.004). Survival ratios are normalized by cell densities of untreated cultures in corresponding conditions. *N* = 3 for each condition and the survival curve points, reporting mean ± SEM. In the final timepoint of the survival curve for 0.1 mg/ml lactose, two of the replicates were below the level of detection; the remaining replicate value is plotted.

The existence of growth heterogeneity in high lactose cultures raises the question of whether some growth-arrested cells function as persisters (slow- or non-growing cells that tolerate antibiotic treatment). Emergence of bacterial persister cells is typically attributed to the action of toxin-antitoxin systems [35, 47]. However, starvation-induced (p)ppGpp signaling [48], entry into stationary phase [49], or cell-cell signaling [50] can also induce persister formation, suggesting that the underlying mechanisms may be diverse. To our knowledge, persister formation from excessive intracellular metabolic activity has not been shown. To determine if persisters arise preferentially in cultures with high lactose concentrations, we measured the kinetics of cellular killing in ampicillin following growth at low (0.1 mg/ml), moderate (1.5 mg/ml) and high (50 mg/ml) lactose concentrations (Fig 4a). To estimate the efficiency of killing, we subsequently (post-treatment) grew cells in the absence of lactose, which should relieve the metabolic burden, allowing surviving culturable cells to seed colonies. Consistent with the presence of persister cells, killing (estimated from colony forming units, CFUs from the media) was biphasic with time. Namely, the first phase of fast killing up to ~6 hours was distinguishable from the second phase of slower killing beyond 6 hours in all conditions. This second phase of killing is caused by persister cells. The second phase of killing in 50 mg/ml lactose had significantly slowed death compared to 1.5 mg/ml. Our results also suggest enrichment of persisters in 0.1 mg/ml lactose, consistent with starvation-induced persister formation [48, 51]. We also grew acclimatized cultures in a range of lactose concentrations with or without the antibiotic doxycycline [52] or ampicillin and counted colony forming units (CFUs) from growth on LB medium as a measure of cell survival after 20 h. Following antibiotic treatment, we found significantly enriched colony formation in populations grown at higher lactose concentrations (Fig 4b), indicating increased antibiotic tolerance. We conclude that conditions that lead to the production of growth-arrested subpopulations also enrich for antibiotic tolerant and persister cells.

### A generalized cellular growth framework based on timescales of molecular events

Our proposed model for metabolism-induced growth arrest represents a limit where toxic side effects slow cellular growth more quickly than shifts in gene expression or detoxification by other factors, such as LacA [53, 54], can alleviate the toxicity. We now introduce a generalized framework for the effects of molecular-scale events on bacterial growth dynamics. From this framework, we derive criteria for the effects of molecular subnetworks on population growth with different timescales.

**Fig 5:**
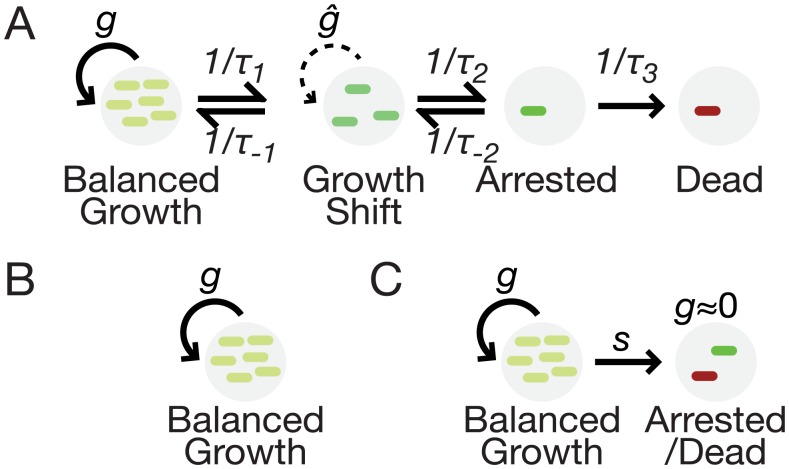
A framework for population growth dynamics in the presence of metabolic risk. (A). Growth conditions, gene expression, replication, and regulatory factors determine timescales for switching between different types of growth. On a characteristic timescale *τ*_*1*_, cells stochastically switch from balanced growth (*g*) to a condition of rapidly changing growth rate (*ĝ*) and escape on a timescale of *τ*_*-1*_. Growth arrest arises from the growth shift state on a timescale of *τ*_*2*_. Escape from growth arrest permits cells to resume growth on a timescale of *τ*_*-2*_, or die on a timescale of *τ*_*3*_. (B) In the limit of large *τ*_*1*_ or small *τ*_*-1*_, populations have classical balanced growth. (C) In the limit of small *τ*_*1*_ and *τ*_*2*_ with large *τ*_*-1*_ and *τ*_*-2*_, the metastable population model holds.

Fig 5a illustrates the generalized framework. It contains four states of bacterial growth: (*i*) balanced, exponential growth; (*ii*) a transient state of unbalanced growth, with growth rates undergoing a change; (*iii*) viable growth arrested cells; (*iv*) dead cells. Characteristic timescales for switching between states are indicated. Various limits on the timescales give well-known types of population growth. For example, as (1/τ_1_)/(1/τ_-1_) → 0, we arrive at balanced exponential growth (Fig 5b). For (1/τ_2_)/(1/τ_-2_) → ∞, we arrive at the metastable population dynamic underlying our model above as well as typical toxin-antitoxin systems. Other growth conditions that may exist as limits of this framework are stationary phase or biofilms where (1/τ_-1_) → 0, and diauxic shifts where 1/τ_1_ is transiently larger than other parameters.

In short, our model of metabolism-driven metastable population dynamics exists as a special-case limit of a larger growth framework. It appears to apply to the *E*. *coli* B REL606 strain grown in lactose. How frequently different parameter limits reflect the effects of different gene regulatory networks remains to be determined. Homeostatic feedback by metabolites could also reduce toxicity by raising the growth arrest threshold (e.g. via PTS IIAGlc feedback to LacY [55]) or eliminating it altogether (S1 Text). For instance, the *E*. *coli* strain K-12 MG1655 does not exhibit the same pattern of dependence of growth rate on lactose concentration (S7a Fig). Further, high lactose concentrations do not produce detectably more persister cells in K-12 compared to a lower lactose concentration (S7b Fig). Via comparisons of the strains, we identify a number of potential mechanisms that could leave the B REL606 strain prone to this effect (S1 Text, S7 Fig, S1 Data). A lack of oxygen in the growth conditions did not appreciably contribute to the effect, as indicated by qualitatively similar results obtained from selected lactose concentrations grown in well-aerated flasks (S9 Fig). No difference in pH between the growth media of the strains was observed in flask or microplate growth (S10 Fig).

## Discussion

In predicting and characterizing the effects of metabolic costs and benefits on bacterial populations at single-cell resolution, our results indicate that metabolic pathways have physiological effects far beyond their metabolite-processing roles. In particular, specific conditions may allow certain metabolic pathways to determine the uniformity or heterogeneity of cellular growth rates. Our results raise the clinically important possibility that some antibiotic persisters are intrinsic consequences of threshold-crossing metabolic events in growing cells.

The effects we found depend on a set of network characteristics common to most, or perhaps all, metabolic pathways: enzyme saturation, toxicity, and a limitation to the benefit that can be derived from pathway products. Another set of criteria for our results suggest how robustness to heterogeneity may form: benefit and toxicity must take effect faster than compensatory regulatory or enzyme-kinetic mechanisms can rescue cells on the trajectory toward growth arrest (Fig 5), and some types of homeostatic feedback may reduce or eliminate the effect (S1 Text). Our experiments provide strong evidence that metastable growth driven by metabolic thresholds is possible in bacterial populations. We see no reason why this conclusion should be limited to any particular species or pathway, but rather its generality is limited by the types of molecular mechanisms present and the criteria for the effect identified in S1 Text.

Questions about the mechanism of metastable population dynamics remain. What is the direct cause of cellular growth arrest? It is possible that off-target toxicity in our experiments, not directly emerging from intermediate production, changes growth rates after LacY is saturated. Our analysis suggests that such an effect would drive a pattern of uniformly reduced growth rates (S1 Text), unlike what we observed experimentally. We chose to test our phenomenological models of toxicity in lactose because of the well known lactose killing effect [26], assuming that permease-related toxicity would be the cost. However, our models predict the same metastable dynamics even with minor toxicity that could arise from intracellular metabolite buildup. It is thus possible that intracellular lactose or downstream metabolite processing contributes to the cost of excess lactose in the media. A comparison between an *E*. *coli* strain subject to the effect (B REL606) with one that is less sensitive to it (K-12 MG1655) explores three possible types of differences that could underlie the effect: amino acid substitutions, changes in gene regulation, and differences in cell size (S1 Text, S7 Fig, S1 Data). Our analysis does not conclusively rule out any of them, but reveals more extensive differences in gene regulatory sequences than amino acid sequences. It also shows that cell size differences could lengthen the timescale of metabolite buildup for a limited range of kinetic parameters. Finally, persister cell enrichment is largely attributed to toxin-antitoxin systems. We cannot rule out that the effects of metabolite excess percolate to TA systems that ultimately induce growth arrest.

With each of these mechanisms, and possibly others, being candidates for driving growth heterogeneity, what is the physiological makeup of the growth arrested pool of cells? We found enrichment of PI+ dead cells and antibiotic persisters in our study. Other phenotypes of non-growing cells may be present as well. For instance, rapid onset of growth arrest may freeze protein concentrations at non-steady-state levels that originated from gene expression noise.

We conclude that single-cell resolution computational and experimental studies are an indispensable tool for understanding how metabolism drives cellular growth. Competing timescales of metabolite kinetics and gene regulation appear to be of central importance for determining population growth dynamics. The relevance of our findings may extend beyond metabolic pathways as well, to other systems governed by supply and demand.

## Methods

### Population growth experiments

This study used *E*. *coli* REL606 *lacI*^−^(or its ancestral *lacI*^wt^) transformed with Tn7∷*P*_lacO1_GFP (Kan^R^) as described previously [44], and *E*. *coli* K-12 MG1655 (Coli Genetic Stock Center). Cultures were started in LB medium (BioWorld) from -80°C storage, grown for 12–15 hours in a shaking 37°C incubator (VWR 1575, Sheldon Manufacturing, Inc.), and resuspended 1:100 in Davis Minimal medium (DM; Difco) supplemented with thiamine along with concentrations of lactose as described in the main text. Resuspended cultures were grown in 96-well non-cell culture-treated flat bottom plates (Falcon) for 24 hours to allow them to physiologically acclimate to the prevailing environment. After acclimation, the cultures were resuspended into fresh 96-well plates containing DM with identical sugar concentrations, 1:100 for flow cytometry and 1:1000 for growth measurements in an incubating microplate reader (BioTek Eon). Representative growth curves are shown in S8 Fig.

The flow cytometry cultures were grown for 5–7 hours in a shaking 37°C incubator, diluted 1:5 in plain DM, and run through a Beckman Coulter MoFlo XDP flow cytometer with Hoechst stain to trigger read events. To assay cell death, PI (1 μg/ml) was introduced to cultures, they were incubated for ten minutes, and the fraction of PI+ cells was quantified using a Beckman Coulter MoFlo XDP. Flow cytometry files were converted to plain text format in R [56], and filtered to eliminate cells more than 0.5 standard deviations around the mean forward and side scatter in linear coordinates. The scatter distributions were relatively tight, so this gate had the effect of eliminating outliers. A very small number of additional outlier points were filtered for having extremely high fluorescence (between 0 and 40 points per condition, maximally 0.13% of the data points; the threshold was being greater than 6-fold higher than the mean fluorescence in 0.1 mg/ml lactose). We computed average fluorescence from the distributions by finding the means of the gated data of 3 culture replicates, and then computing mean ± standard error of the means. The flow cytometry files are available from Flow Repository, ID: FR-FCM-ZZLJ.

Flow cytometric analysis of cells in each lactose concentration presented have three biological replicates each (including independent measurements of GFP and PI stain). The overall trend in GFP expression has been observed in three separate sets of experiments and PI staining has been observed in two separate sets of experiments.

Microplate reader cultures were grown for 24 hours at 37°C with continuous shaking and a reading of OD_450_ every two minutes. We computed growth rates from resulting growth curves in early exponential phase using a log-linear model fit in R, and finding mean ± standard error of the growth replicates. Growth rate experiments have three biological replicates per condition. The overall growth response trend for growth in lactose in *E*. *coli* B REL606 has been measured at least three times (with 3–4 biological replicates each). The overall growth response trend for growth in lactose in *E*. *coli* K-12 MG1655 has been measured twice (with 3 biological replicates each).

### Well-aerated flask experiments

To confirm that low oxygen levels in the plate reader did not qualitatively change our conclusions, we repeated flow cytometry for cultures in representative lactose concentrations (2.5 mg/ml and 50 mg/ml) that were grown in well-aerated 150 ml flasks in 10 ml of culture (S9A Fig). Colony forming units and OD_450_ readings were taken periodically to determine growth curves and set a time to sample for flow cytometry (S9B and S9C Fig). We selected 4h post-inoculation for flow cytometry, and carried it out as described above.

### pH measurement

To rule out the possibility that acidification of the culture media underlies the higher growth heterogeneity in B REL606 than K-12 MG1655, cultures of the two strains were grown in 50 mg/ml lactose in well-aerated 150 ml flasks in 10 ml of culture, and pH of samples measured with a Mettler-Toledo pH meter (S10 Fig). To check for pH differences in the 96-well microplate setup, we carried out a plate reader experiment in 50 mg/ml lactose with both strains out to 7.5 hours, representative of the final time used to fit the growth curves. At that time, we pelleted the cells in a centrifuge, and measured the spectral properties of supernatant from B REL606 and K-12 MG1655 treated with the pH-indicating dye phenol red (30 μg/ml) using an Agilent Cary 60 UV-Vis spectrophotometer with a quartz cuvette. Changes in the pH level are reflected in the spectral peak near 460 nm (S10 Fig). At this wavelength, the absorbance difference between the strains is non-significant (*p* = 0.27; *N* = 3 for each strain).

### Time-lapse microscopy and image processing

Cultures were acclimatized in 1 ml cultures as described above in 14 ml polypropylene tubes (Falcon), resuspended 1:1000, and grown for an additional 5–6 hours. At this time, the cultures were resuspended to an OD_600_ of approximately 0.005 and added to an ONIX microfluidic plate (CellAsic model B04A). Cultures were grown for 18–24 hours with a heated manifold maintaining 37°C, a constant flow of fresh air over the culture chamber, and constant perfusion (1 psi) of fresh media on a temperature-stabilized 100x oil immersion objective in a Nikon Ti1000 microscope. Every two minutes, the imaged field was algorithmically autofocused and images taken in bright field, at 460 nm (green fluorescence) and at 565 nm (red fluorescence). At the end of the experiment, DM with the same concentration of lactose containing propidium iodide (1 μg/ml) was perfused for 5–10 minutes to stain dead cells, and images of various locations in the chamber were then captured. Individual color channels in images in Fig 3 were adjusted for brightness; PI stained cells appear as bright red or yellow, depending on the relative PI and GFP levels.

Time-lapse microscopy has been repeated 2–3 times for some of the conditions to confirm the existence of the patterns observed.

### Antibiotic persister detection

*E*. *coli* cultures were started and acclimatized in various concentrations of lactose as described above. After stabilization, the cultures were resuspended into identical lactose concentrations containing either 32 μg/ml doxycycline [52], 100 μg/ml ampicillin, or a control treatment without antibiotic. For the antibiotic tolerance assay, these cultures were grown for 20 hours and plated onto LB plates without antibiotic selection at 1x, 10x, 100x, and 1000x dilutions (and up to 10^7^-fold dilution for untreated cultures). For the ampicillin kill curves, samples were taken at the indicated time points and diluted appropriately for accurate CFU counts on LB plates.

In the antibiotic tolerance assay, each 96-well culture plate had a single lactose dose with ampicillin, doxycycline, or no antibiotic controls. Persister timecourses have three biological replicates per time point and the experiment has been done twice for B REL606 and once for K-12 MG1655. Survival ratios after 20h have been measured twice for each strain (3 biological replicates) to confirm the pattern. Survival ratios were calculated from raw data by matching between treated (*x*_*i*_) and untreated (*y*_*i*_) replicates originating from the same culture, and calculating statistics from the ratio log_10_(*x*_*i*_/*y*_*i*_) calculated for each replicate.

### Simulations, data processing and mathematical analysis

Analysis of the numerical results, mathematical models, and data processing not otherwise specified were done in Mathematica versions 8 or 9 (Wolfram Research).

To determine statistical trends in the data, subsets of data points were subjected to linear or nonlinear regression and the resulting ANOVA statistics reported using the LinearModelFit or NonlinearModelFit function in Mathematica.

To assess the effects of intrinsic biochemical noise on predicted fitness in the simple metabolic pathway, we extended a previously published model of this pathway that included reactions capturing transcription, translation, substrate-enzyme interactions and catalysis, and concentration-dependent molecular dilution at a constant growth rate [39]. Simulation models were constructed in Copasi 4.8 (Build 35) [57]. Stochastic simulations were performed with StochKit 2.0.1 [58] on a computational cluster using detailed mass action propensities for chemical reactions and custom propensities for dilution and degradation reflecting growth feedback effects. The model includes complex propensity functions that capture the effects of changing growth rates on dilution and transcription rates [45] as outlined in Table 1.

For each condition, we ran 10,000 simulations. For each simulation, we set initial conditions equal to the mean field steady state for the given parameter set and ran time trajectories for 8,000 s of simulation time each. Population growth rates were calculated as the mean growth rate across the trajectories. Because the trajectory did not include any contribution from cell division, parameter sets resulting in a high probability of crossing the threshold into growth arrest resulted in virtually all trajectories entering the growth arrest state; as a result, conditions with very high simulated metabolite production rates result in predictions of virtually no growth. Changing the trajectory time would thus have the effect of changing the fraction of cells entering growth arrest. The chosen time of 8000 s demonstrates the principle on a timescale relevant to cell growth and minimizes the effects of the initial condition on the final result. An exemplary file used for stochastic simulations is presented in S2 Data.

### Genetic comparisons of *E*. *coli* B REL606 and K-12 MG1655

To identify possible underlying mechanisms of growth arrest in B REL606 in high lactose, we analyzed genetic differences between that strain and K-12 MG1655, which is less prone to the effect (S1 Text). We first identified 65 proteins involved in lactose processing or events directly downstream, including glycolysis, galactose degradation (the Leloir pathway), and other enzymes or transporters that result in the production or degradation of lactose, glucose, or galactose (listed in S1 Data). For each protein, we retrieved the amino acid sequence for each strain from UniProt, and identified coordinates of regulatory regions for each gene in each strain. Regulatory sequences were identified based on the well-annotated K-12 MG1655 strain, and putative regulatory regions of B REL606 genes were selected to encompass intergenic regions up to the neighboring operon. Databases of *E*. *coli* gene and protein sequences were used in the analysis as described in greater detail in S1 Text [59–62].

Each pair of amino acid or nucleotide sequences was aligned with ClustalW2 and the score recorded in S1 Data. Alignment scores were not penalized for having unaligned regions. To determine if the regulatory DNA sequences were more alike than a randomized sequence, we used a resampling procedure to generate 1000 pairs of randomized sequences for each actual sequence, with nucleotides for each sequence randomly sampled with replacement from its corresponding actual sequence. We calculated alignment scores for the randomized pairs, used them to create a numerical score distribution, and calculated from that the probability that the alignment score for each regulatory region was more alike than random.

## Supporting Information

S1 Text Mathematical, statistical, bioinformatic, and time-lapse microscopy analysis. (PDF)

S1 Fig Models and growth phase diagrams for metabolic pathways with relaxed properties shows sufficient conditions for three growth regimes. (A) Pathway with low toxicity metabolite that does not stimulate cellular growth. Regimes I and III are possible depending on metabolite production. (B) Pathway completely absent toxicity from the metabolite. Non-growing Regime III does not exist. (C) Pathway where the consuming enzyme never saturates, eliminating Regime III. (D) Pathway with growth stimulation modeled in the form of a saturating Michaelis-Menten type of function that imparts diminishing benefit for higher concentration preserves the sharp transition between Regimes I and II and Regime III, but blurs the surface between Regimes I and II. For this surface, *f*_*gr*_ = 0.9 is shown.
(EPS)

S2 Fig Existence of steady states as a function of critical parameters from model rescaling. (A) For consumption flux below the demand threshold *δ*, changing values of *R*, the ratio of maximal enzyme activities, can eliminate the steady state. (B) Likewise, changes in *Θ*, the scaled toxicity threshold, can eliminate the steady state. (C, D) The same behavior is confirmed for scaled parameters *R* and *Θ* with consumption flux above the demand threshold *δ*.
(EPS)

S3 Fig Population dynamics and fitness around a critical point that controls growth rate. (A) A population of cells grows at rate *g* and switches irreversibly to the non-growing phase at rate *s*. The fraction of growth arrested cells is a function of CDF(*x*) with *x* = *V*^+^/*V*^-^ the ratio of mean fluxes. (B) Normalized growth rates (color bar; gray denotes < 0) depend on demand for substrate (*δ*) and ratio of mean fluxes *x* = *V*^+^/*V*^-^.
(EPS)

S4 Fig Population growth rates of (A) *lacI*^−^ and (B) wild-type strains in varying concentrations of lactose are virtually identical. Data in (A) are taken from Fig 2, shown for comparison with the wild-type strain.
(EPS)

S5 Fig A heterogeneous growth rate model explains measured mean fluorescence values of a constitutive reporter in high lactose concentrations. (A) Fit of a uniform growth fluorescence model to the experimental data. Dashed line, calibration curve (S21). Each black point represents a growth rate-fluorescence pair in a different lactose concentration. Red numbers label lactose concentration for select conditions. (B) Fit of a heterogeneous growth model to the experimental data and least-squares values as a function of free parameters in the heterogeneous growth model. Experimentally measured growth rate means at each lactose concentration were used to predict the fluorescence. (C) Correlation of fluorescence models with measured data: uniform model, -0.080; heterogeneous model, 0.833. (D) Family of heterogeneous model parameter values with sum of squares; blue region corresponds to minimal values. Red point in blue region denotes parameters used for panels B-C. *p*_*arr*_, protein concentration in terms of fluorescence (AU); *s*, rate of cells switching to growth arrest (/h).
(EPS)

S6 Fig Characteristics of constitutive fluorescent reporter distributions in various concentrations of lactose. (A) Replicates of smoothed fluorescence distributions showing the moving average of three logarithmic bins. The mode clearly shifts lower for increasing lactose concentrations. (B) Changes in skewness and excess kurtosis, or “peakedness,” of the distributions as lactose concentration increases. Both measures increase significantly at lactose doses increase (ANOVA F = 36.0735, *p* < 10^−6^ for skewness and F = 29.5816, *p* < 10^−6^ for excess kurtosis; N = 3 for each lactose dose), suggesting increasing heterogeneity of growth.
(EPS)

S7 Fig Identification of possible underlying mechanisms of lactose-induced growth heterogeneity in *E*. *coli* B REL606 by comparison to *E*. *coli* K-12 MG1655. (A) Comparison of growth rates between the strains in varying lactose. (B) Kinetics of cell death under antibiotic treatment in low and high lactose concentrations reveals no detectable change in persister formation in *E*. *coli* K-12 MG1655, unlike B REL606. Concentrations represent lactose in Davis minimal media. (C) Alignment scores of amino acid sequences for 65 genes involved with lactose catabolism or downstream events reveals high conservation. Three proteins have notably lower alignment scores (< 98): UDP-D-glucose:(glucosyl)LPS α-1,3-glucosyltransferase (*waaO*), Lipopolysaccharide glucosyltransferase I (*waaG*), and Glucose-1-phosphate thymidylyltransferase 1 (*rfbA*). (D) Alignment scores of regulatory nucleotide sequences for 65 genes involved with lactose catabolism or downstream events reveals variable conservation. (E) Probabilities that the regulatory alignment scores are lower than random reveal possible regulatory divergence for many of the genes. (F) In a mathematical model, time difference in a 10% change in cell volume for metabolite levels to reach a threshold (1000 molecules/(cell volume)) starting from zero metabolite demonstrates that larger cell volumes could open a window for compensatory regulation to prevent growth arrest. Numbers indicate *K*_*m*_ (molecules/(cell volume)) of the metabolite-consuming enzyme for the corresponding line. Region to the right of the dashed line does not have a realizable steady state in the model. Parameters used: *δ* = 500; *V*_*max*_[*B*] = 1; *θ* = 100; *g*_*max*_ = 0.0006.
(EPS)

S8 Fig Representative OD_450_ growth curves used to measure population growth rates in a shaking plate reader. Curves were cut off before and after deviating from exponential growth without deviations, and fitted to determine the population growth rate.
(EPS)

S9 Fig Growth patterns are unchanged in well-aerated flask cultures. To ensure that hypoxia in plate reader growth did not change our conclusions, we repeated flow cytometry and growth experiments with otherwise identical protocols in 10 ml cultures grown in 150 ml flasks in the strain B REL606 *lacI*^*–*^. Blue, 2.5 mg/ml lactose. Red, 50 mg/ml lactose. Mean fluorescence was normalized to the largest mean value. *N* = 3 biological replicates. A. Mean (μ) and coefficient of variation (CV = σ/μ) of constitutive GFP after 4 h growth. *N* = 3 biological replicates. B. Growth experiment colony forming units (CFU). C. Growth experiment OD450, with media blanks subtracted. *N* = 3 biological replicates. Error bars, SEM.
(EPS)

S10 Fig Growth patterns are not correlated with acidic metabolic byproducts. A. Optical density (OD450) time course of the cell culture medium, with media blanks subtracted. B. Time course of acidity (measured as pH) for the growth medium. C. UV-Vis spectra of phenol red-stained supernatants from cultures grown for 7.5 h. Differences in pH would be reflected in absorbance differences at the 560 nm peak, but absorbance is not significantly different between the strains (*p* = 0.27). *N* = 3 biological replicates. Error bars, SEM. Blue, K-12 MG1655. Red, B REL606 *lacI*^−^. All culture media in these experiments contained 50 mg/ml lactose.
(EPS)

S1 Movie Time-lapse microscopy of *Escherichia coli* perfused with 0.1 mg/ml lactose. Composite images of bright field, green, and red channels (bright field is blue in the RGB colorspace of the video) for time-lapse microscopy of *lacI*^−^*E*. *coli* with *P*_lacO1_GFP perfused by a constant lactose concentration of 0.1 mg/ml. Frames were captured every two minutes.
(MOV)

S2 Movie Time-lapse microscopy of *Escherichia coli* perfused with 1 mg/ml lactose. Composite images of bright field, green, and red channels (bright field is blue in the RGB colorspace of the video) for time-lapse microscopy of *lacI*^−^*E*. *coli* with *P*_lacO1_GFP perfused by a constant lactose concentration of 1 mg/ml. Frames were captured every two minutes.
(MOV)

S3 Movie Time-lapse microscopy of *Escherichia coli* perfused with 2 mg/ml lactose. Composite images of bright field, green, and red channels (bright field is blue in the RGB colorspace of the video) for time-lapse microscopy of *lacI*^−^*E*. *coli* with *P*_lacO1_GFP perfused by a constant lactose concentration of 2 mg/ml. Frames were captured every two minutes.
(MOV)

S4 Movie Time-lapse microscopy of *Escherichia coli* perfused with 50 mg/ml lactose. Composite images of bright field, green, and red channels (bright field is blue in the RGB colorspace of the video) for time-lapse microscopy of *lacI*^−^*E*. *coli* with *P*_lacO1_GFP perfused by a constant lactose concentration of 50 mg/ml. Frames were captured every two minutes.
(MOV)

S1 Data Detailed data comparing 65 proteins involved in lactose processing or events directly downstream from *E*. *coli* strains K-12 MG1655 and B REL606. (XLSX)

S2 Data File for stochastic simulations of the simple metabolic pathway with growth feedback. (XML)

S3 Data Experimental data plotted in figures. (GZ)
