# Supplementary material for: Cellular Growth Arrest and Persistence from Enzyme Saturation
Source: PLoS Comput Biol. 2016 Mar 24;12(3):e1004825. doi: 10.1371/journal.pcbi.1004825 (PMC4820279; doi:10.1371/journal.pcbi.1004825)
Supplement: S1 Text — (PDF) [file pcbi.1004825.s001.pdf]

Supplementary Text for:

Cellular growth arrest and persistence from enzyme saturation

J. Christian J. Ray, Michelle L. Wickersheim, Ameya P. Jalihal, Yusuf O. Adeshina, Tim F. Cooper, and Gábor Balázsi

Text S1

Analytical derivation of growth phases

We explore the effects of various fitness cost and benefit functions for a metabolite M produced by enzyme A and consumed by enzyme B with traditional Michaelis-Menten type kinetics (i.e., reversible binding of substrate to enzyme with an irreversible catalysis step). The assumptions of our models are as follows: (i) we assume a single-celled, symmetrically dividing organism and use parameter values representative of bacteria; (ii) for the mean-field models, we use a quasi-steady-state assumption for gene expression states. The second assumption implies that the effects of metabolite concentrations and fluxes around critical thresholds affect cellular growth rates faster than gene expression changes can compensate. This assumption is tested, and found to be reasonable, by stochastic simulations (Fig. 2a-c) and by analysis of protein kinetics during growth arrest presented below. We further explore relaxing this assumption in the main text.

The model follows a metabolite produced and degraded by enzymes A and B, at rates V^+ and V^- respectively, and also subject to dilution from cellular growth that depends on the emergent growth rate. We write the model as follows:

$$\dot{M} = V^+ - V^-(M) - g_{max} f_{tox} f_{stim} M \tag{S1}$$

for an arbitrary metabolite M. The last term represents dilution of M from growth. It is calculated as the product of the maximum attainable growth rate g_{max}, metabolite toxicity function f_{tox}, and a function of growth stimulation by the downstream product f_{stim}. We detail specific cases below.

Sources of growth stimulation and repression from metabolic pathways

The consumption flux V^- of the simple metabolic pathway can stimulate growth by providing downstream metabolites. Here, an important distinction arises between different levels of demand on the pathway – that is, what level of flux may be attained before growth is no longer stimulated by further activity. In some contexts, many alternative routes exist for carbon flux and no single pathway is necessary for growth. Then the contribution of a single pathway to growth may be minimal, and demand for that pathway is low. On the other hand, in many cases growth is rate-limited by flux through a single pathway (e.g., the initial catabolic steps specific to the only carbon source present in minimal media). To capture growth stimulation in the model, we assume a maximum hypothetical growth rate g_{max}, and take the consumption flux V^- normalized by the demand δ:

$$f_{stim} = a + \phi(V^-, \delta) \tag{S2}$$

where a is the contribution to growth from alternate pathways. For the remainder of this analysis we consider the effects of a single essential metabolic pathway (e.g. single sugar uptake and catabolism in minimal media) and assume $a = 0$. We introduce the piecewise growth-stimulation function

$$\phi(V^-, \delta) = \begin{cases} \dfrac{V^-}{\delta}, & V^- \le \delta \\ 1, & V^- > \delta \end{cases} \tag{S3}$$

that increases with V^- below the demand threshold, and becomes constant above it.

Growth repression may arise from a number of sources, including excess metabolite concentration [13, 24], side effects of enzyme activity (such as proton symport from sugar permeases [33]), or costs of protein expression. Increasingly many studies

implicate metabolite production, as opposed to protein expression [25] , in the costs associated with metabolism. We consider two models for toxicity: stress from proton symport activity (or any byproduct of metabolite production) and toxicity from the metabolite itself. In either case we have

$$f_{tox} = \frac{\theta}{\theta + [toxin]} \tag{S4}$$

where the toxin is proton or metabolite concentration and θ is an inverse measure of toxicity (i.e. smaller θ implies larger effect of the toxin on growth repression).

Model: toxicity from metabolite

The model presented in the main text depicts the metabolite as the direct growth inhibitor. This model has a simple form, with a single dynamic equation:

$$\dot{M} = V^+ - \frac{V_B BM}{K_B + M} - g_{max}\phi(V^-, \delta)\frac{\theta}{\theta + M}M \tag{S5}$$

where B is the concentration of enzyme catalyzing V^-; this model has steady state value

$$\begin{gathered}\hat{M} = \frac{\delta\left[-V_B\theta B + V^+\left(K_B + \theta\right)\right] + \sqrt{\Gamma}}{2\left[V_B B\left(\delta + g_{max}\theta\right) - \delta V^+\right]} \\ \Gamma = 4\delta K_B\theta V^+(V_B\delta B + V_B g_{max}\theta B - \delta V^+) \\ + (V_B\delta\theta B - \delta K_B V^+ - \delta\theta V^+)^2\end{gathered} \tag{S6}$$

for $V^- = \frac{V_B BM}{K_B + M} < \delta$, and

$$\begin{gathered}\hat{M} = \frac{-[\theta(V_B B + K_B g_{max}) - V^+(\theta + K_B)] + \sqrt{\Delta}}{2(V_B B + g_{max}\theta - V^+)} \\ \Delta = [\theta(V_B B + K_B g_{max}) - V^+(\theta + K_B)]^2 + 4V^+K_B\theta(V_B B + g_{max}\theta - V^+)\end{gathered} \tag{S7}$$

for $V^- > \delta$. We arrive at a critical surface beyond which no physically realizable steady state exists given by

$$\begin{aligned} &V_B B\left(\delta + g_{max}\theta\right) - \delta V^+ = 0, \quad V^- < \delta \\ &V_B B + g_{max}\theta - V^+ = 0, \quad V^- \geq \delta \end{aligned} . \tag{S8}$$

An additional critical surface represents the transition between Regime I (where flux capacity is at or below demand; $V^- \leq \delta$) and Regime II (where flux capacity exceeds demand; $V^- > \delta$) in Figure 1b. We find this transition at the point

$$V^- = \frac{V_B BM}{K_B + M} = \delta . \tag{S9}$$

In terms of demand versus supply (δ vs V^+ as in Figure 1), the transition between Regime I and Regime II is given by:

$$V^+ = \delta + g_{max} \frac{K_B}{\frac{V_B B}{\delta} + \frac{K_B}{\theta} - 1} . \tag{S10}$$

We deduce from these conditions an analogy to a triple point between Regimes I, II and III:

$$(\delta, V^+) = (V_B B, V_B B + g_{max}\theta) . \tag{S11}$$

Sufficiency conditions for the emergence of each growth phase

The critical surfaces between each growth phase emerge from specific, and separable, effects in the model. We can see this by individually eliminating growth inhibition, growth stimulation, and enzyme saturation from the model to show that the complete three-phase diagram depends on all three phenomena. The models with these relaxed

assumptions may also be representative of some pathways; e.g. if a pathway has no effect on cellular growth rates.

Without growth stimulation, only the threshold between phases I and III remains (Fig. S1a):

$$V^{+} = g_{max}\theta + V_B B \tag{S12}$$

while without inhibition, the growth arrested phase is eliminated (Fig. S1b). In a model without saturation, the threshold between phases I and III is eliminated as well (Fig. S1c). Therefore, saturation of V^{-} and growth inhibition from the pathway together are sufficient for the growth arrested phase to emerge.

Model: Alternate source of growth inhibition

An alternative source of toxicity from excess V^{+} lies in side effects of activity for the enzyme *A* (that catalyzes V^{+}) producing the metabolite. In the case of catabolic systems, non-PTS, non-ABC transporters derive their motive force from proton symport [59]. If H^{+} symport upsets osmotic balance, perhaps growth benefit from V^{-} more than offsets the metabolic cost of correcting the imbalance [33]. If this effect underlies the toxicity of metabolic imbalance in the *lac* operon, it is likely that the same effect occurs in many Major Facilitator Superfamily transporters [60]; for instance, D-arabinose, L-fucose, β-D-galactose, L-arabinose, and L-rhamnose are all imported by proton symporters in *E. coli* [59].

Extending the model where the metabolite is toxic (Eq. (S1)), we have:

$$\begin{aligned} \dot{M} &= V^{+} - V^{-}\left(M\right) - g_{max} f_{tox}^{H^{+}} f_{stim} M \\ [\dot{H}^{+}] &= V^{+} - f_{tox}^{H^{+}} f_{stim} \cdot [H^{+}] \end{aligned} \tag{S13}$$

with

$$f_{tox}^{H^+} = \frac{\theta}{\theta + [H^+]}. \tag{S14}$$

We find a critical surface given by

$$\delta = \begin{cases} \dfrac{g_{max}\theta V_B B}{V^+}, & g_{max}\theta V_B B \le V^+ \\ g_{max}\theta, & g_{max}\theta V_B B > V^+ \end{cases} \tag{S15}$$

so that, qualitatively, a similar effect occurs as with direct toxicity of the metabolite but there is now a maximum demand permitting cellular growth, $\delta = g_{max}\theta$ (Fig. 1b). Intuitively, this means that growth stimulation from the pathway can be limited by proton symport or other side effects of metabolite production and still produce three phases of cellular growth.

Model: Alternate form for growth stimulation

If we take a different model for the threshold of maximal growth stimulation from the pathway, e.g. a diminishing returns model captured by a Hill function $H\left(V^-\right) = \frac{\left(V^-\right)^h}{K_I^h + \left(V^-\right)^h}$ (with the demand set by parameter K_I in this case), the exact position in the threshold exists in a limiting case for large V^-. In practice, the difference between the critical surface and its threshold becomes smaller than the blurring effects of chemical and gene expression noise at reasonable quantities of V^-; we plot the threshold at which $H\left(V^-\right) = 0.9$ in Fig. S1d.

In conclusion, the emergence of the three discrete growth phases depend on the existence of an inhibition process and a upper-bounded growth stimulation process, but not on their specific underlying mechanisms.

Model: Off-target source of toxicity

An additional form of toxicity could arise from off-target effects, such as high concentrations of lactose slowing growth by an unknown mechanism without the necessity for intermediate production. Using the same approach as above, a cost-benefit model for this case would have a constant M, the same form of benefit function as Eq. (S3) (assuming that growth is proportional to metabolite uptake rate), and the same toxicity function as Eq. (S4) with M as the toxic agent. Then the net benefit has the form

$$f_{stim} f_{tox} = \phi \frac{\theta}{\theta + M} = \begin{cases} \dfrac{V_{max} \theta M}{\delta \left(K_m + M \right) \left(\theta + M \right)}, & \dfrac{V_{max} M}{K_m + M} < \delta \\ \dfrac{\theta}{\theta + M}, & \dfrac{V_{max} M}{K_m + M} \geq \delta \end{cases} \tag{S16}$$

where V_{max} and K_m are the kinetic parameters for lactose (or other metabolite) uptake. While this is a plausible toxicity mechanism in general, it lacks the critical transition into a specifically growth-arrested state, and therefore does not represent a likely mechanism for metastable population growth dynamics.

Model: Homeostatic feedback

The effects observed in the no-feedback case could be avoided by homeostatic feedback inhibition. We therefore explored if an effect of feedback could be to eliminate the singularity in the steady state M that is diagnostic of the growth-arrested regime, analogous to Eq. (S8). Three cases are informative of the possible effects of feedback:

one where the metabolite M inhibits the production flux V^+, and two where the product of metabolite transformation by V^- inhibits either V^+ or V^-. Inhibitory feedback on V^+ could be from an arbitrary number of steps forward; feedback by P is representative of this case. For simplicity, because we are analyzing the effect of toxicity rather than benefit, we can consider the case where there is no further benefit from higher pathway flux ($V^- > \delta$) without loss of generality.

Feedback inhibition model extensions of the metabolite toxicity model in Eq. (S1) with the standard mathematical form of competitive inhibition are:

$$\begin{aligned} V^+_{Pfb} &= \frac{V^+}{K_i + P} \\ V^-_{Pfb} &= \frac{V_B B M}{K_B + M + \frac{K_B P}{K_i}} \\ V^+_{Mfb} &= \frac{V^+}{K_i + M} \end{aligned} \tag{S17}$$

where K_i is the dissociation constant of the inhibitor. Here, we have introduced a new variable, P, the product of the flux V^-. P also has a consumption flux given by $k_u P$ and growth-mediated dilution as with M. The full solutions to these models are bulky and unnecessary for determining the existence of the growth arrest threshold; it suffices to determine the existence of singularities in the denominator. The case of product (P) feedback to V^+ has a singularity for the solution of the system for $\hat{M}$ given by

$$0 = \left(K_i k_u + V_B B\right)\left(g_{max}\theta + V_B B\right) - k_u V^+ . \tag{S18}$$

Similarly, for product feedback to the flux V^-, we have

$$0 = V^+ - V_B B - g_{max}\theta . \tag{S19}$$

Therefore, feedback to the step producing a putative toxic metabolite can increase the threshold, but does not eliminate it, while feedback to the step consuming the toxic metabolite does not affect the threshold.

The result is different if the metabolite inhibits its own production step. Here, the analogous term in the denominator of $\hat{M}$ is

$$0 = V_B B + g_{max}\theta . \tag{S20}$$

This threshold is only met in the trivial case of zero pathway flux and no growth, and it cannot be surpassed. Incidentally, we observe that feedback inhibition from *M*, but not *P*, eliminates the singularity because there is not a saturation step that buffers M from the extent of feedback. To demonstrate this observation, we considered the case of feedback to V^+ by *P* with a non-saturatable flux V^- as in Fig. S1c. The resulting threshold for the existence of the growth arrest regime is given by $0 = V_B B$, again showing the regime to be eliminated in non-trivial cases.

We conclude that homeostatic negative feedback inhibition can eliminate the toxified growth-arrest regime as long as there is not a saturating process. Thus, direct inhibition of toxic metabolite production by itself may be an important homeostatic step to eliminate the effect. Feedback from further downstream does not eliminate the regime, but it may make entry into the toxified state less likely.

Model rescaling reveals critical parameters for identifying growth phases

The steady state equation for $V^- = \dfrac{V_B BM}{K_B + M} < \delta$ in the no-feedback model can be written in the form

$$\frac{V^+}{V_B B} = \frac{\frac{M}{K_B}}{1+\frac{M}{K_B}}\left[1+\frac{g_{max}K_B}{\delta}\frac{\frac{\theta}{K_B}\frac{M}{K_B}}{\frac{\theta}{K_B}+\frac{M}{K_B}}\right]. \tag{S21}$$

By introducing the dimensionless parameters $R = \frac{V^+}{V_B B}$; $g = \frac{g_{max}K_B}{\delta}$; and $\Theta = \frac{\theta}{K_B}$, as well as the dimensionless variable $m = \frac{M}{K_B}$, we obtain

$$R\frac{1+m}{m} = 1+g\frac{\Theta\, m}{\Theta + m}. \tag{S22}$$

These equations indicate that 3 critical parameters define the behavior of this system. The first parameter $R = \frac{V^+}{V_B B}$ is the ratio of maximum enzyme activities (or the ratio of metabolite production to maximum metabolite consumption). The second parameter $g = \frac{g_{max}K_B}{\delta}$ relates the maximum growth rate and the demand to the threshold of enzyme *B*. The third parameter $\Theta = \frac{\theta}{K_B}$ is the toxicity threshold scaled by the Michaelis-Menten constant of enzyme *B*.

The steady state metabolite level will correspond to the point where the functions

$$f_L(m) = R\left(1+\frac{1}{m}\right) \tag{S23}$$

and

$$f_R(m) = 1+g\frac{\Theta m}{\Theta + m} \tag{S24}$$

intersect each other. The "enzyme effect" function $f_L(m) = V^+/V^-$ is monotone decreasing from infinity when $m \to 0$ to R when $m \to \infty$, while the "growth effect" function $f_R(m)$ is monotone increasing from 1 when $m \to 0$ to $1 + g\Theta$ when $m \to \infty$. Such a pair of functions can intersect each other at most once, provided that the following condition is satisfied: $1 + g\Theta > R$. This is exactly the relationship (S8), confirming that phase transitions can be visualized as the functions $f_L(m)$ and $f_R(m)$ missing each other (Fig. S2a-b; R=2, g=1, Θ=2).

The steady state equation for $V^- = \dfrac{V_B BM}{K_B + M} \geq \delta$ can be written in the form:

$$\frac{V^+}{V_B B} = \frac{\frac{M}{K_B}}{1 + \frac{M}{K_B}} + \frac{g_{max} K_B}{V_B B} \frac{\frac{\theta}{K_B} \frac{M}{K_B}}{\frac{\theta}{K_B} + \frac{M}{K_B}}. \tag{S25}$$

Once again, we introduce the dimensionless parameters $R = \dfrac{V^+}{V_B B}$; $G = \dfrac{g_{\max} K_B}{V_B B}$; and $\Theta = \dfrac{\theta}{K_B}$, as well as the dimensionless variable $m = \dfrac{M}{K_B}$, to obtain:

$$R \frac{1+m}{m} = 1 + G\Theta \frac{1+m}{\Theta + m}. \tag{S26}$$

The same considerations apply as above, except now the "growth effect" function takes the form $f_R(m) = 1 + G\Theta \dfrac{1+m}{\Theta + m}$, which can be monotone increasing from $1 + G$ when $m \to 0$ to $1 + G\Theta$ when $m \to \infty$ and $\Theta > 1$ and monotone decreasing between the same values when $\Theta < 1$. When $\Theta = 1$, the function $f_R(m) \equiv 1 + G$ becomes a constant.

The condition for a steady state to exist is again $1+G\Theta > R$, exactly as in (S8) (Fig. S2c-d).

Reporter protein kinetics as an indicator of growth rate

In our experiments, we use a chromosomally-integrated, constitutively expressing GFP gene as a sensor for growth rates in single cells. We claim that the pattern of mean fluorescence observed is consistent with heterogeneous growth including a fraction of cells transitioning into growth arrest.

Previous work on growth-rate-dependent gene expression suggested that transcription rates, translation rates, gene dosage, protein dilution, and cell volumes depend on growth rate while translation rates and mRNA degradation rates do not [45]. The dependence of gene dosage, protein dilution, and cell volumes is approximately linear while transcription rates saturate at high growth rates. A simple homogenous protein concentration model shows the dependence of protein concentration on these factors: $p = \frac{\gamma \alpha_m \alpha_p}{V \beta_m \beta_p}$ where γ is gene dosage, V is cell volume, α_m and α_p are mRNA and protein synthesis rates, and β_m and β_p are mRNA and protein degradation rates. Implementing the dependence of each parameter on growth rate g, we have $\alpha_m \approx \frac{s_{\alpha_m}}{K_{\alpha_m} + g}$, $\gamma \approx s_g g$, $V \approx s_V g$, and $\beta_p \approx s_{\beta_p} g$. We can then fit uniform growth protein concentrations (protein/mass) to a simple model:

$$p \approx p_{fit}(g) = \frac{a}{K+g} \tag{S27}$$

where $a = \frac{s_g s_m \alpha_p}{s_V s_{\beta_p} \beta_m}$ and $K = K_{\alpha_m}$.

The heterogeneous model is a generalization of this one, dependent on the population dynamics of growth arrest. We consider a population model with two states: an exponentially growing (at rate g) sub-threshold population x_L and a growth arrested population x_H. The L → H transition happens at rate s. Then after the initial transient dynamics, the populations are described by the time-dependent equations:

$$\begin{aligned} x_L(t) &= x_{L0}\, e^{(g-s)t} \\ x_H(t) &= \frac{s}{g-s} x_{L0}\, e^{(g-s)t} \end{aligned} \tag{S28}$$

where x_{L0} and is the population of x_L at $t = 0$. The effective population growth rate after a sufficiently long time is $g_{net} = g - s$. Our heterogeneous fluorescence model is then

$$p_{het} = \frac{g_{net}}{g_{net} + s} \cdot p_{fit}(g_{net} + s) + \frac{s}{g_{net} + s} \cdot p_{arr} \tag{S29}$$

where p_{arr} is the effective mean protein concentration in growth-arrested cells, in the same fluorescence units as the experimental GFP measurements.

Our model in Figure 1, combined with simulations shown in Figure 2c-d, predict an increasing fraction of cells entering growth arrest with higher lactose concentrations. To estimate parameters in the uniform growth model, we excluded the bottom five lactose concentrations (≤ 0.5 mg/ml) where growth measurements were noisy and evidently widespread cell death from starvation occurred (Fig. 3a), fit the next five lowest lactose doses (0.6 mg/ml through 1.0 mg/ml) and predicted the remaining fluorescence values with the uniform growth model (Fig. S5). The calibration curve, equation (S27), is plotted in Fig. S5a (dashed line). Clearly, the uniform growth model does not capture

mean fluorescence at lactose concentrations expected to cause a large amount of growth arrest (i.e. > 2 mg/ml; Fig. S5a).

We next sought a plausible fit for the heterogeneous growth model. Using the previous homogenous growth fit parameters, we are left with two additional parameters: p_{arr} and s. The patterns of CV (Fig. 3b), microfluidic cultures (Fig. 3c) and thickness of the fluorescence distribution tails (Fig. S6, discussed below) as lactose concentrations increase suggests a progressive model for arrested cell fluorescence, where intensity in growth-arrested cells is correlated with lactose concentrations in the growth-arresting regime. The goal of this fit is to choose parameters that minimize the term

$$\Lambda = \sum_i \left(X_i - p_{het,i} \right)^2$$
$$p_{het,i} = \frac{g_{net,i}}{g_{net,i} + s} \cdot p_{fit}(g_{net,i} + s) + \frac{s}{g_{net,i} + s} \cdot p_{arr} \cdot [\mathrm{lac}]_i \quad \text{(S30)}$$

where each parameter with an i subscript is given by the experimental data, X_i being fluorescence values and $[\mathrm{lac}]_i$ denotes the concentration of lactose correponding to fluorescence and growth measurements i. Note that the apparent noisiness of the model arises from the growth rate data input and does not reflect additional model complexity. The progressive model provides an improved fit (min Λ; Fig. S5b), with a correlation of 0.833 between predicted and experimental fluorescence points compared to virtually no correlation in the uniform model (correlation = -0.080; Fig. S5c).

What is the minimal value of s that provides a least-squares fit to the data? If the family of least-squares fits (Fig. S5d) does not permit $s = 0$, then the heterogeneous growth model is a better fit to the experimental results. If, on the other hand, there is a value for parameter p_{arr} that allows $s = 0$ for a least-squares fit, we cannot rule out

homogenous growth rates. Plotting Λ for each possible pair of (s, p_{arr}) in the heterogeneous growth model, we find a unique value of s minimizing Λ at 0.030 /h (Fig. S5d). With this best fit, we find a predicted minimal fraction of growth-arrested cells of $\phi = s/g = 0.090$ (i.e. 9 percent) in 50 mg/ml lactose. We conclude that the experimentally observed mean fluorescence values are consistent with our growth arrest model.

Complete fluorescence distributions for three replicates at several lactose doses (Fig. S6a), along with the average skewness and excess kurtosis of the distributions (Fig. S6b), show further evidence that population dynamics differ as lactose doses increase. Notably, the excess kurtosis, a measure of the shape of the distribution, becomes increasingly positive (leptokurtotic), indicating a more peaked distribution with fatter tails as the concentration of lactose is increased.

Analysis of population dynamics around a growth-rate-determining critical point

To estimate growth rate characteristics necessary to maintain a growing population, we consider the population dynamics model introduced above in Equation (S28). This model shows that an exponentially growing subpopulation constantly producing non-growing cells maintains exponential growth at a rate reduced by the switching rate: $g_{net} = g - s$ (inset, Fig. S3a). The key to positive growth rates is maintaining $g > s$. To find optimal population growth, we maximize the quantity $g_{net} = g - s$.

Understanding how the trade-off between metabolic cost and benefit determines the overall growth rate calls for a model to simulate how g and s are affected by the underlying biochemical kinetics. The parameters of the distribution for M are dominated by the balance between production and consumption fluxes $x = \left\langle V^+ \right\rangle / \left\langle V^- \right\rangle$ because of

the large quantitative effect of the saturation point for V^-. As this model measures the cumulative fraction of the population that is growth arrested, we have $s = k_s \mathrm{F}_s(x)$ with rate parameter k_s determined by the rate of M buildup once the consuming enzyme is saturated. F_s is the cumulative distribution function of s (Fig. S3a; note that $\mathrm{F_s}(x) = \mathrm{CDF}(x)$). The ratio x also determines the average benefit to the population, assumed to be increasing with x up to the level of demand δ. A possible phenomenological description of the benefit is $g = \begin{cases} k_g \dfrac{x}{\delta} & x \le \delta \\ k_g & x > \delta \end{cases}$. The intuitive interpretation of the rate constant k_g is as the growth rate for optimal cells in the absence of the critical transition.

In one simple case, the distribution of x is exponential with cumulative distribution function $\mathrm{F}_s(x) = 1 - e^{-x/\beta}$; mean and standard deviation are given by β. Because g monotonically increases with x while s monotonically decreases with it, the optimal growth rate is in the range $g_{net} \le \delta$ and is given by the relation

$$0 = \frac{\partial g_{net}}{\partial x} = \frac{\partial (g - s)}{\partial x} = \frac{k_g}{\delta} - \frac{k_s}{\beta} e^{-\frac{x}{\beta}} . \tag{S31}$$

The predicted optimal growth rate is at the point $x^* = \beta \ln\left(\frac{k_s \delta}{k_g \beta}\right)$ or $x^* = \delta$, whichever is smaller. Here we expect a growth arrested population fraction of

$$\phi = \begin{cases} \dfrac{\frac{k_s \delta}{k_g \beta} - 1}{\ln\left(\frac{k_s \delta}{k_g \beta}\right)}, & x^* < \delta \\ \dfrac{k_s}{k_g} - \dfrac{\beta}{\delta}, & x^* = \delta \end{cases} .$$

Thus, for a population of cells with an exponentially distributed population of flux ratios, we have shown that a fraction of cells are expected to be growth arrested. This can be seen as a cost-benefit result. The maximal net benefit, g_{net}, is improved by pathway activity up to a point, then reduced by it. The existence of a positive fraction of growth-arrested cells with the optimal point $x^* > 0$ shows that some cells are guaranteed to be growth arrested with the irreversible growth transition model.

Comparison of response to lactose in two *E. coli* strains reveals possible mechanisms underlying growth heterogeneity

The heterogeneous response to high lactose concentrations in the strain B REL606 is interesting given that growth defects in a constant lactose environment require genetic modifications to the K-12 strain *lac* operon [33]. We confirmed that there is no apparent growth defect in wild type K-12 MG1655 in lactose concentrations that do cause growth arrest in B REL606 (Fig. S7A), and that conditions enriching persister cells in B REL606 do not do so for K-12 MG1655 (Fig. S7B). The two strains have multiple genetic differences, including notable differences in production of the LPS core [61]. B REL606 also has a smaller volume per cell in many conditions. These observations suggest three possible explanations for differences in toxicity: protein chemistry, gene regulation, and cell volume.

First, amino acid substitutions could result in altered kinetic parameters of *lac* operon proteins or another enzyme downstream of lactose, with amino acid sequence variation between the strains evident in enzymes and regulators relating to lactose catabolism or downstream steps, including glycolysis, the Leloir pathway (galactose degradation), and other mechanisms of glucose and galactose consumption or efflux. To assess the

plausibility of this explanation, we identified 65 proteins directly relating to lactose, glucose, or galactose, retrieved their amino acid sequences from the UniProt database for each strain, and computed their alignment scores with the program ClustalW2 (Data S1; Fig. S7C). The results show high conservation of the *lac* operon (one amino acid change in the product of *lacA*, a detoxifying enzyme [53]). There is a single non-conserved residue in the Leloir pathway (*galT*, gal-1-P uridylyltransferase) and some non-conserved residues in glycolysis enzymes (products of *ppsA*, *gpmI*/*yibO*, *glpX*, and *phoA*). Changes in other pathways of lactose, glucose, and galactose utilization or transport were evident as well. These include most notably 50% conservation in the product of *waaO* (UDP-D-glucose:(glucosyl)LPS α-1,3-glucosyltransferase) and 90% conservation in *waaG* (Lipopolysaccharide glucosyltransferase I), both involved in previously noted differences in LPS production. Our analysis suggests a small number of possible downstream metabolic steps could have variable kinetic parameters between the strains.

Second, differences in gene regulation or transcript processing may result in enzyme ratios closer to the ultrasensitive threshold in B REL606, increasing the chance of growth arrest. To explore the plausibility of this explanation, we systematically identified regulatory regions for the operons encoding each of the 65 proteins identified above in EcoCyc [59] and BioCyc [62] and aligned them with ClustalW2 (Data S1). The alignment did not penalize for completely non-homologous regions: the alignment scores reflect only regions of homology in the regulatory sequences. This condition reduces the chance of erroneous annotation producing misleading results. The alignment scores indicate perfect alignment between 8 of the regulatory regions regulating 9 genes (*lacZYA*, *yggF*, *glpX*, *setA*, *ybiV*, *gcd*, and *glgC*) and much lower scores for the remaining

(≤ 33; Fig. S7D). To determine how divergent the regulatory sequences are, we used a permutation test, creating 1000 bootstrapped sets of sequences from each regulatory sequence by randomly sampling from the corresponding sequence. We computed a numerical distribution from the 1000 randomized sequence alignment scores, and calculated the probability that the alignment score is higher than random (Fig. S7E). The results show that 19 of the regulatory regions have probabilities less than 50%, opening the possibility of strong divergence in their regulation. Notably, the regulatory region for the *galETKM* operon (most of the Leloir pathway) has a probability of 0.018, strongly suggesting divergent regulation of galactose catabolism between the strains.

Finally, a smaller cell volume could lower the time required for metabolic buildup to surpass the growth arrest threshold, preventing the B REL606 strain from escaping growth arrest, assuming that there is no compensatory regulation that holds the enzyme concentration to be effectively the same. The smaller cell volume in B REL606 may only require a smaller number of molecules required for a metabolite or byproduct to comprise a toxic proportion of the cytoplasm, or there could be a slower rate of efflux caused by smaller cell wall surface area. In this scenario, dilution or efflux of the toxic agent (excess metabolite or metabolic byproduct) is sufficient to prevent toxicity in K-12 MG1655 but not B REL606. Escape could thus happen by permitting sufficient time for regulatory compensation.

To determine if this is a plausible scenario, we performed simulations of metabolite buildup with the ODE model presented in (S5). For each simulation, we scanned metabolite production (V^+) and calculated the time to build up from 0 molecules/(cell volume) to a threshold value of 1000 molecules/(cell volume) with the cell volumes 10%

different. We plotted the difference in time to reach the threshold for a variety of K_m values in the metabolite-consuming enzyme (Fig. S7F). The faster metabolite is produced, the lower the difference in time between cell volumes. However, for production rates close to the threshold for existence of a steady state (given by (S8)), the time to reach the threshold for a 10% increase in cell volume can plausibly reach values long enough to permit downstream gene regulation, as long as the K_m of the enzyme is not too high and the enzyme turnover rate is not exceedingly fast.

In conclusion, we cannot conclusively rule out any of the possible differences between the strains. The most striking difference between them is in gene regulatory sequences, suggesting differences in gene regulation. Nevertheless, cell size differences in a limited range of kinetic parameters could underlie the difference, and key amino acid differences may possibly have large effects despite their relative scarcity.

Time-lapse microscopy of *E. coli* growth in varying lactose concentrations

Movies S1-S4 capture composite images of bright field, green, and red channels (bright field is blue in the RGB colorspace of the video) for time-lapse microscopy of the *lacI*$^-$ strain perfused by constant lactose concentrations (in mg/ml) of 0.1 (Movie S1), 1 (Movie S2), 2 (Movie S3), and 50 (Movie S4). Prior to the last few frames of the videos, propidium iodide-containing medium was introduced and many of the depolarized growth-arrested cells became stained.

Cells entering growth arrest are apparent. Heterogeneity of fluorescence is lower in Movie S1 than in Movies S2-S4. In higher lactose concentrations, many of the brighter cells persist with (apparently slower) growth; this indicates that the slow-down in growth immediately below the critical threshold for Regime III (Fig. 1 and Fig. S2) contributes to

the observed fluorescence heterogeneity. Our models suggest an extended region of growth reduction before reaching the irreversible threshold if proton symport is the relevant toxic effector (Fig. S2) consistent with this effect.
